# Supplementary material for: Pilot Study of the Effects of Chronic Intracerebroventricular Infusion of Human Anti-IgLON5 Disease Antibodies in Mice
Source: Cells. 2022 Mar 17;11(6):1024. doi: 10.3390/cells11061024 (PMC8947551; doi:10.3390/cells11061024)
Supplement: Supplementary file 1 [file cells-11-01024-s001.zip › cells-1590917-supplementary.pptx]

## Slide 1
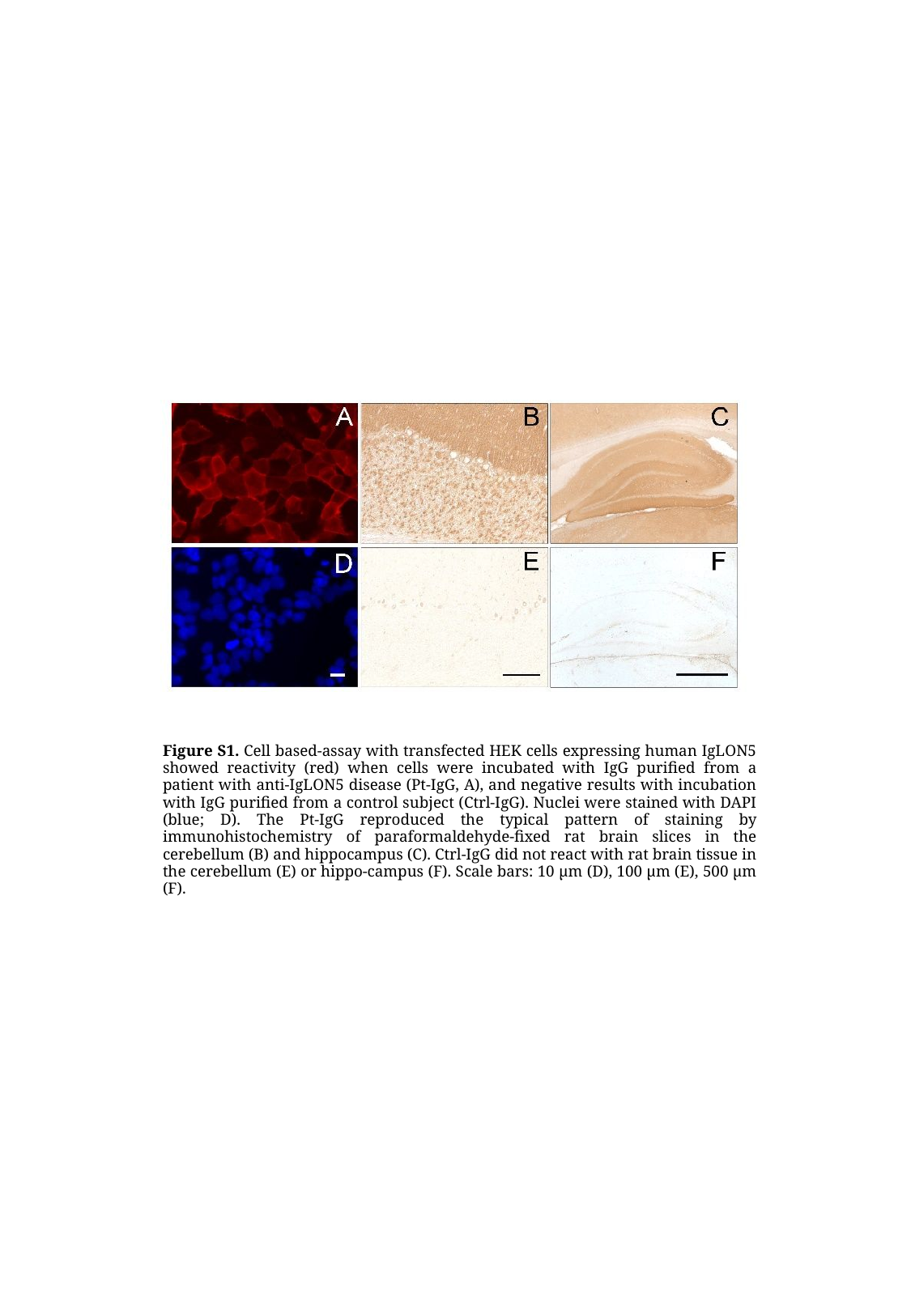

Figure S1. Cell based-assay with transfected HEK cells expressing human IgLON5 showed reactivity (red) when cells were incubated with IgG purified from a patient with anti-IgLON5 disease (Pt-IgG, A), and negative results with incubation with IgG purified from a control subject (Ctrl-IgG). Nuclei were stained with DAPI (blue; D). The Pt-IgG reproduced the typical pattern of staining by immunohistochemistry of paraformaldehyde-fixed rat brain slices in the cerebellum (B) and hippocampus (C). Ctrl-IgG did not react with rat brain tissue in the cerebellum (E) or hippo-campus (F). Scale bars: 10 μm (D), 100 μm (E), 500 μm (F).

## Slide 2
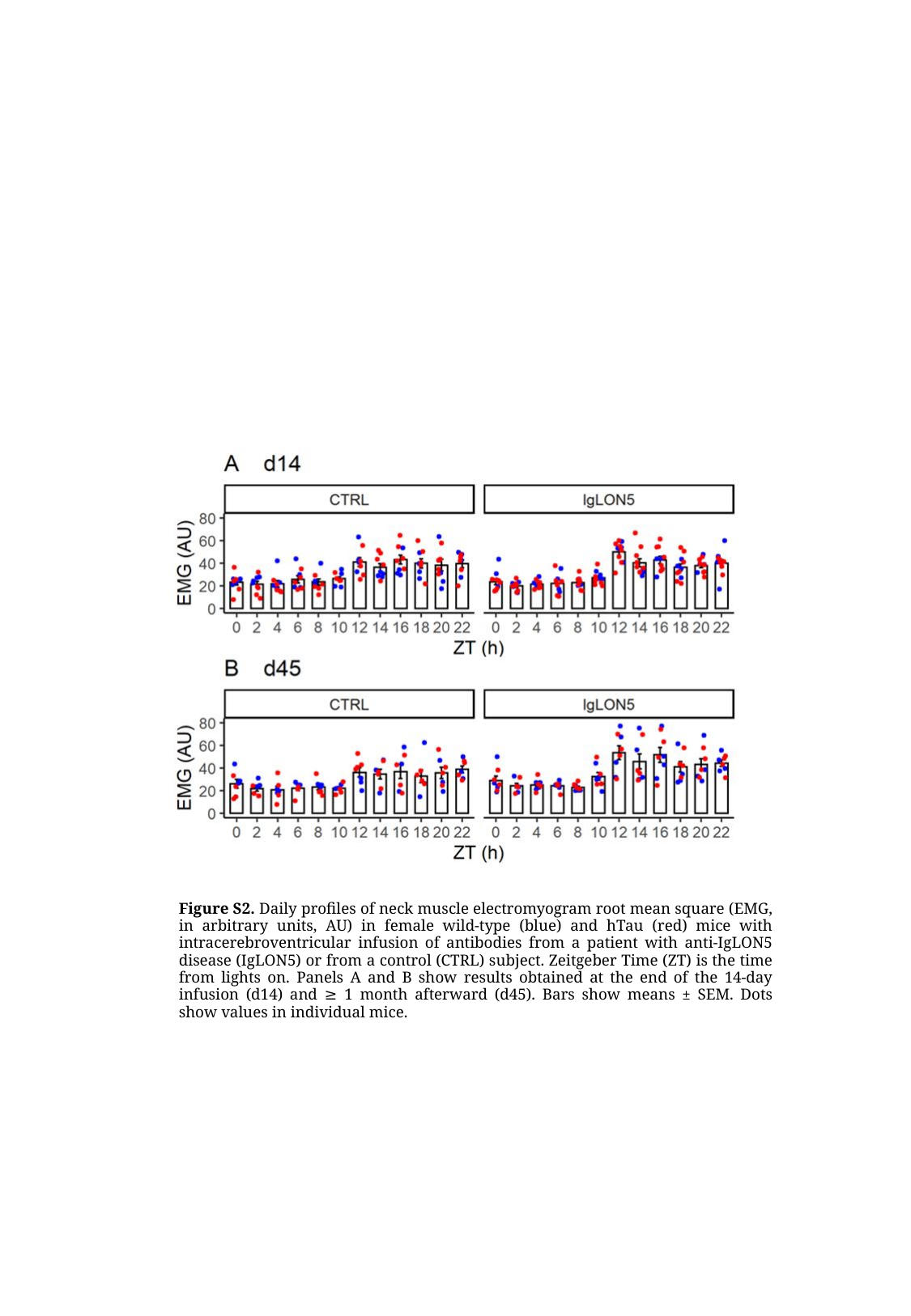

Figure S2. Daily profiles of neck muscle electromyogram root mean square (EMG, in arbitrary units, AU) in female wild-type (blue) and hTau (red) mice with intracerebroventricular infusion of antibodies from a patient with anti-IgLON5 disease (IgLON5) or from a control (CTRL) subject. Zeitgeber Time (ZT) is the time from lights on. Panels A and B show results obtained at the end of the 14-day infusion (d14) and ≥ 1 month afterward (d45). Bars show means ± SEM. Dots show values in individual mice.

## Slide 3
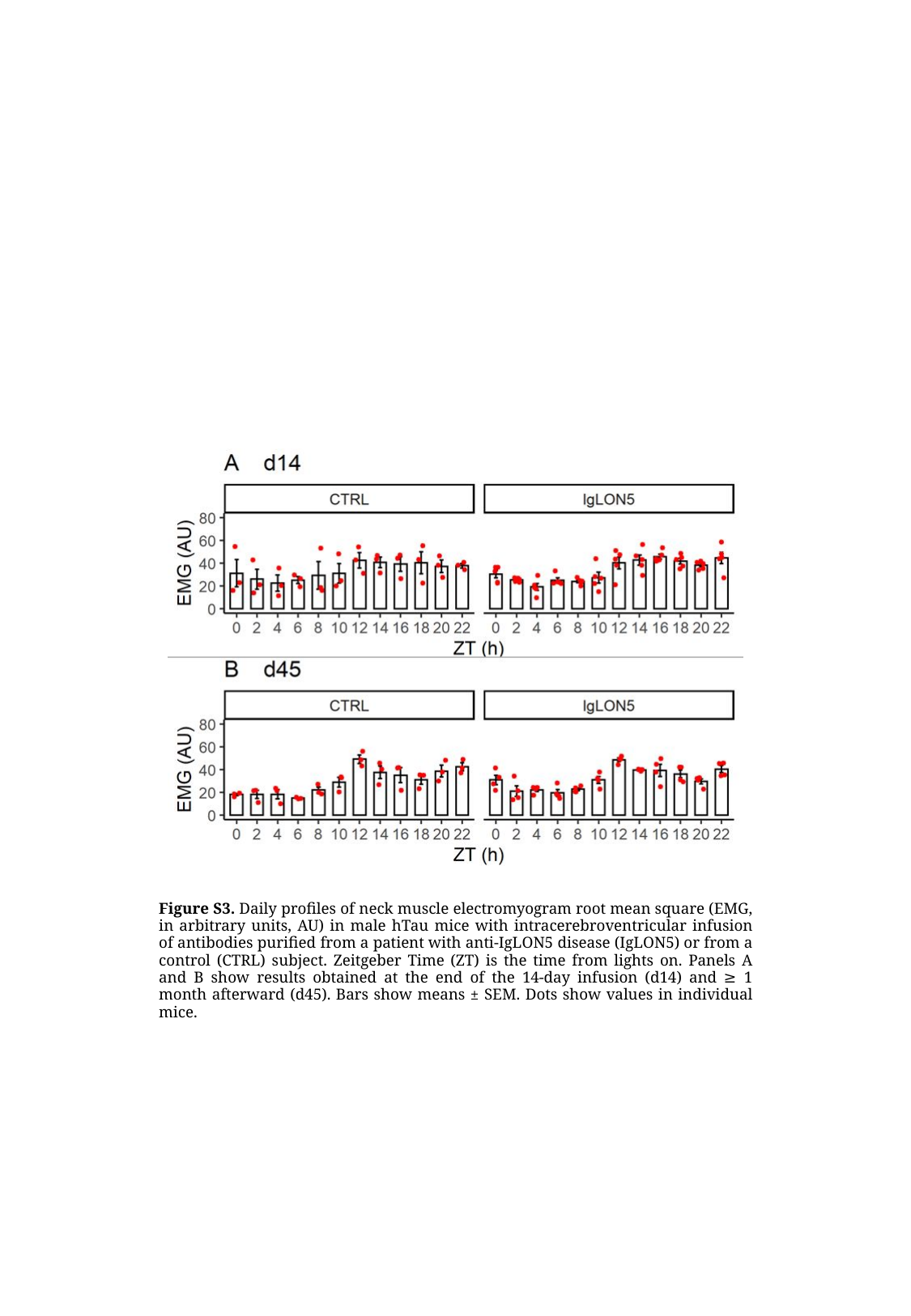

Figure S3. Daily profiles of neck muscle electromyogram root mean square (EMG, in arbitrary units, AU) in male hTau mice with intracerebroventricular infusion of antibodies purified from a patient with anti-IgLON5 disease (IgLON5) or from a control (CTRL) subject. Zeitgeber Time (ZT) is the time from lights on. Panels A and B show results obtained at the end of the 14-day infusion (d14) and ≥ 1 month afterward (d45). Bars show means ± SEM. Dots show values in individual mice.

## Slide 4
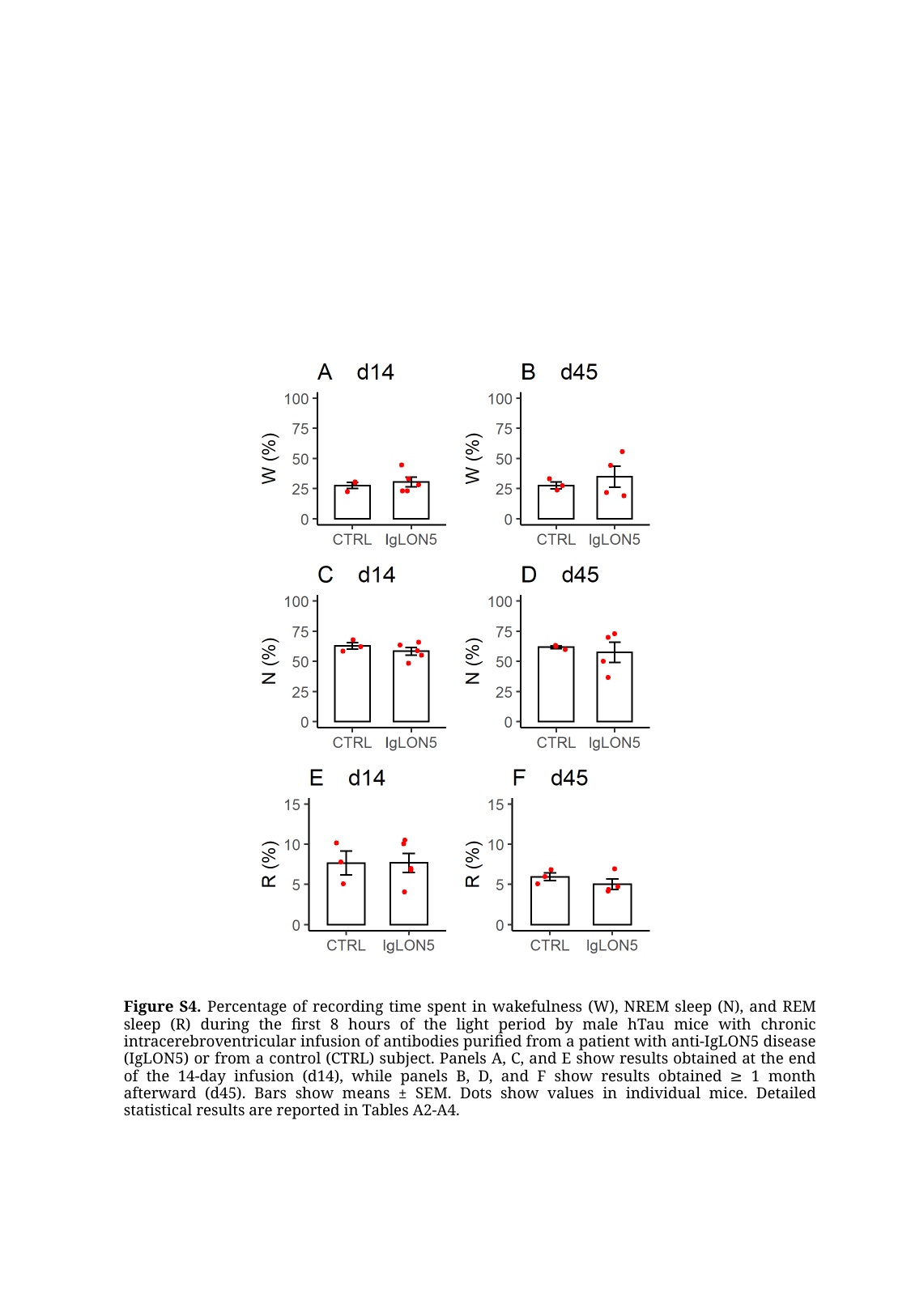

Figure S4. Percentage of recording time spent in wakefulness (W), NREM sleep (N), and REM sleep (R) during the first 8 hours of the light period by male hTau mice with chronic intracerebroventricular infusion of antibodies purified from a patient with anti-IgLON5 disease (IgLON5) or from a control (CTRL) subject. Panels A, C, and E show results obtained at the end of the 14-day infusion (d14), while panels B, D, and F show results obtained ≥ 1 month afterward (d45). Bars show means ± SEM. Dots show values in individual mice. Detailed statistical results are reported in Tables A2-A4.

## Slide 5
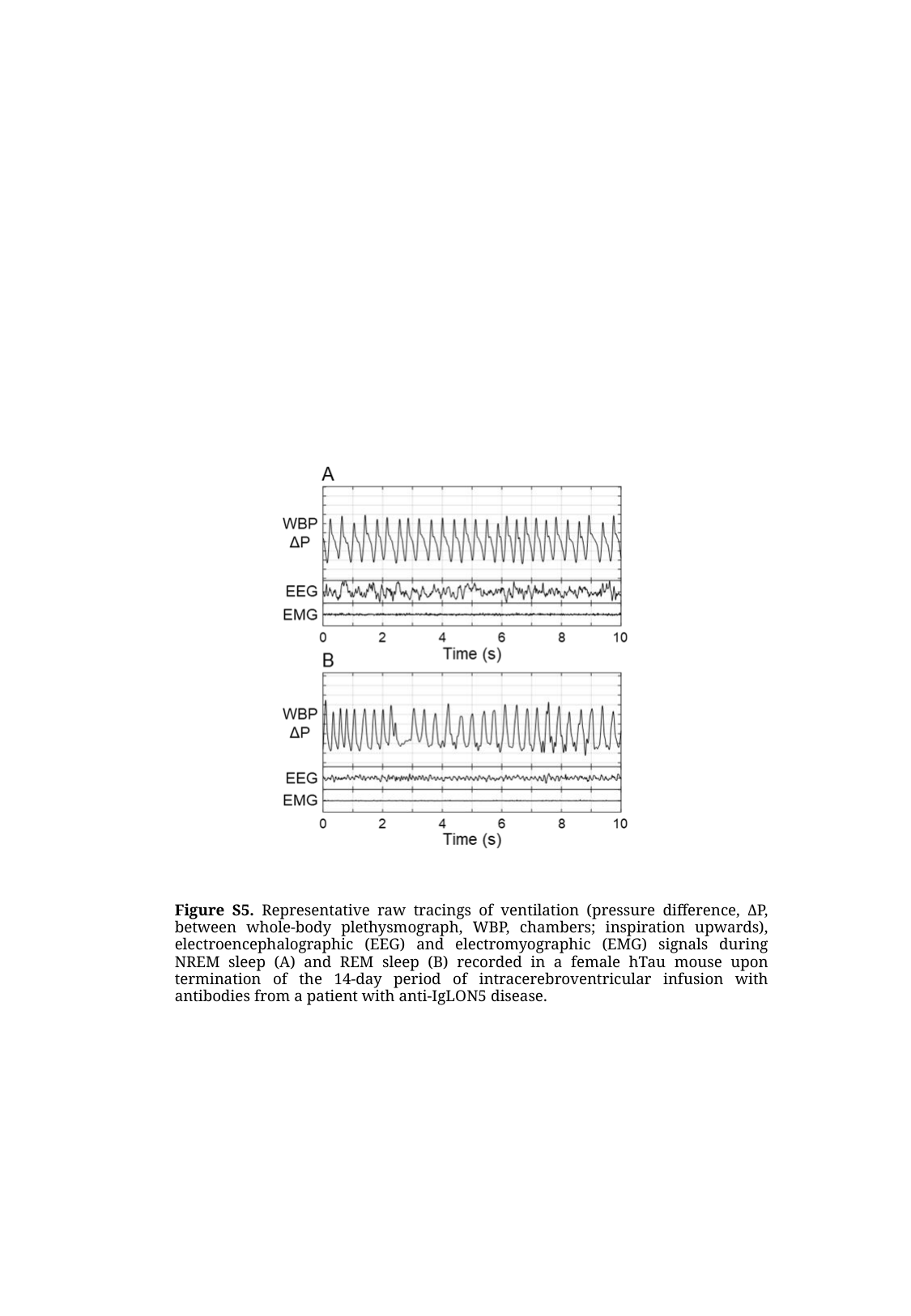

Figure S5. Representative raw tracings of ventilation (pressure difference, ΔP, between whole-body plethysmograph, WBP, chambers; inspiration upwards), electroencephalographic (EEG) and electromyographic (EMG) signals during NREM sleep (A) and REM sleep (B) recorded in a female hTau mouse upon termination of the 14-day period of intracerebroventricular infusion with antibodies from a patient with anti-IgLON5 disease.

## Slide 6
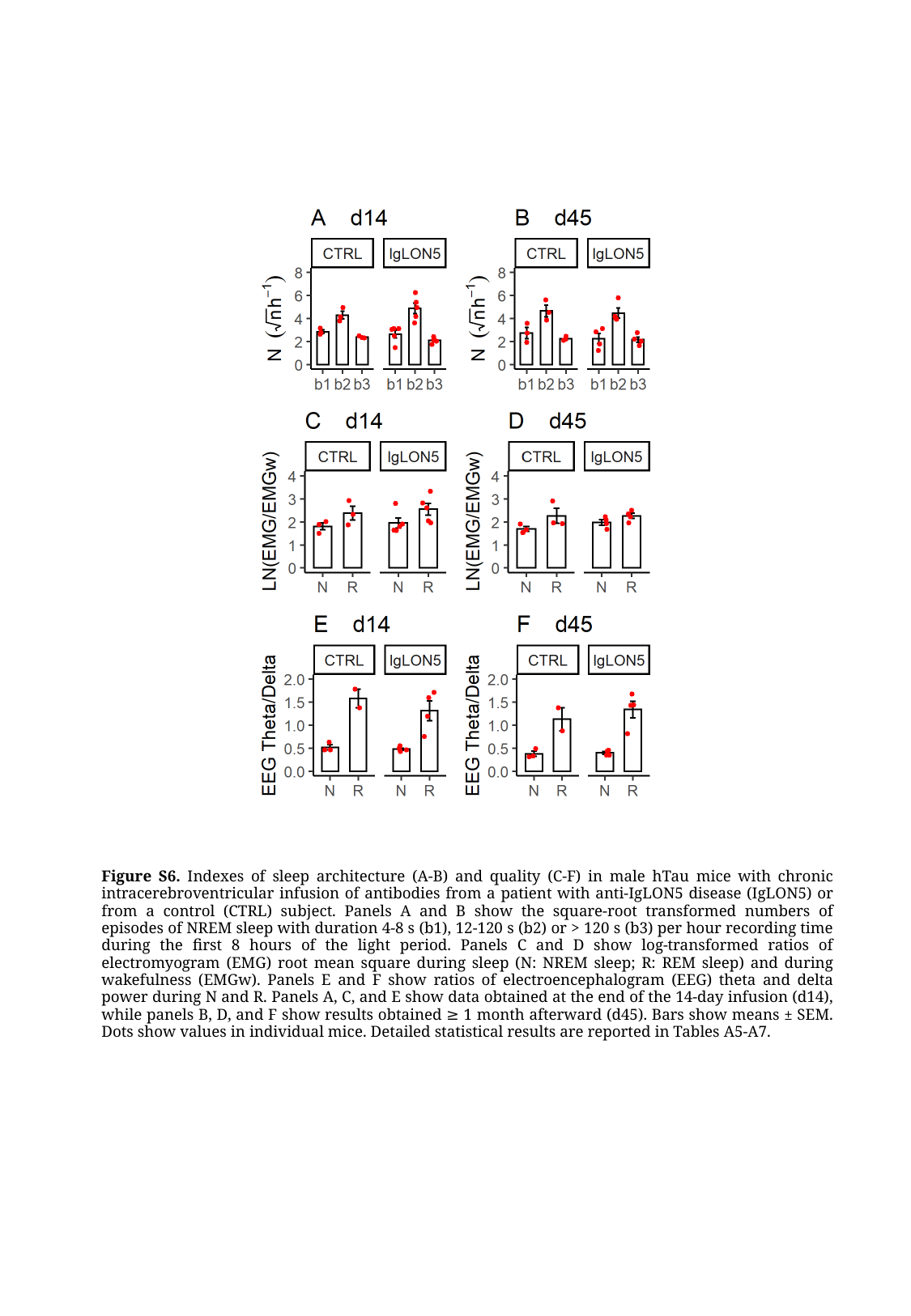

Figure S6. Indexes of sleep architecture (A-B) and quality (C-F) in male hTau mice with chronic intracerebroventricular infusion of antibodies from a patient with anti-IgLON5 disease (IgLON5) or from a control (CTRL) subject. Panels A and B show the square-root transformed numbers of episodes of NREM sleep with duration 4-8 s (b1), 12-120 s (b2) or > 120 s (b3) per hour recording time during the first 8 hours of the light period. Panels C and D show log-transformed ratios of electromyogram (EMG) root mean square during sleep (N: NREM sleep; R: REM sleep) and during wakefulness (EMGw). Panels E and F show ratios of electroencephalogram (EEG) theta and delta power during N and R. Panels A, C, and E show data obtained at the end of the 14-day infusion (d14), while panels B, D, and F show results obtained ≥ 1 month afterward (d45). Bars show means ± SEM. Dots show values in individual mice. Detailed statistical results are reported in Tables A5-A7.

## Slide 7
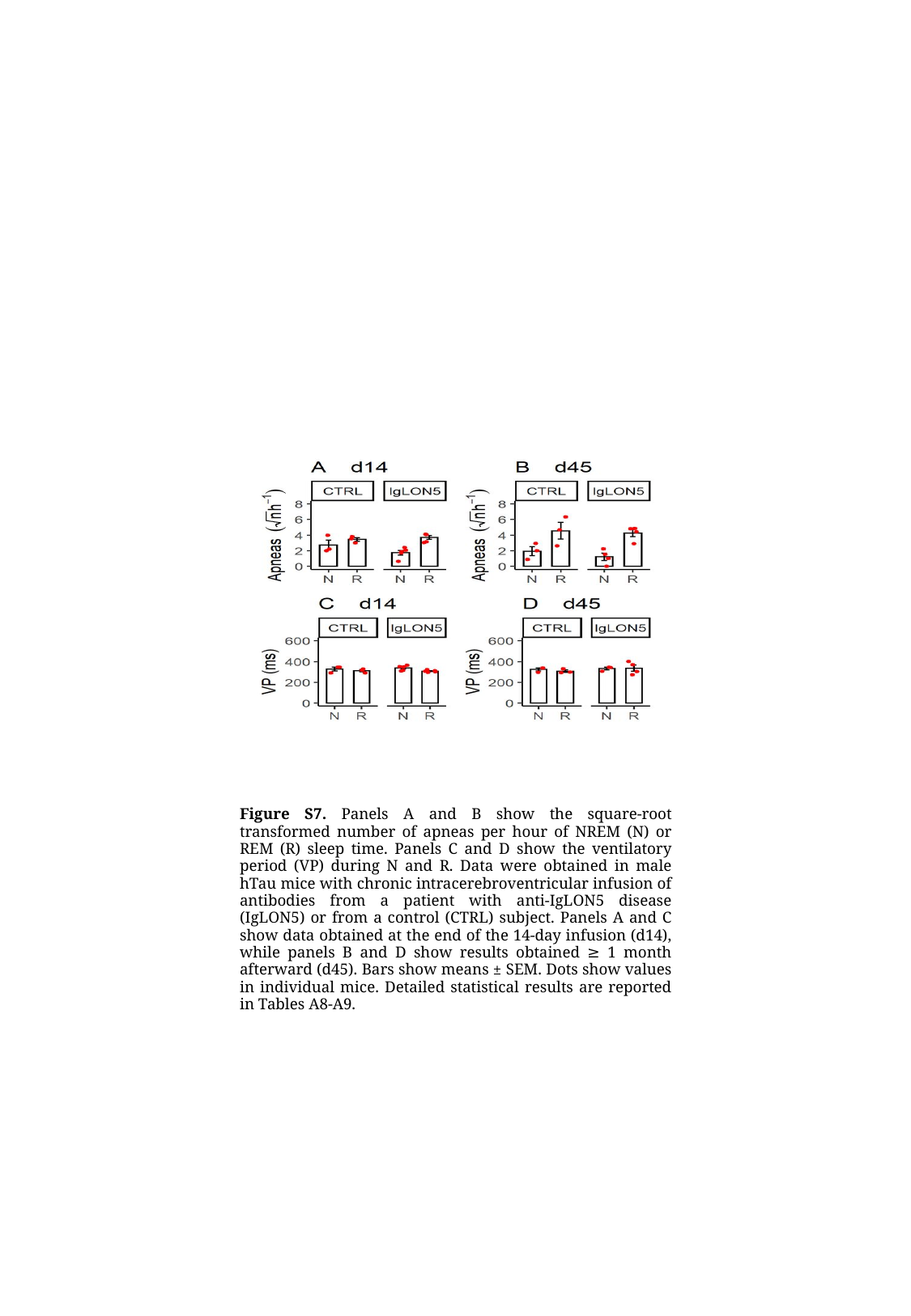

Figure S7. Panels A and B show the square-root transformed number of apneas per hour of NREM (N) or REM (R) sleep time. Panels C and D show the ventilatory period (VP) during N and R. Data were obtained in male hTau mice with chronic intracerebroventricular infusion of antibodies from a patient with anti-IgLON5 disease (IgLON5) or from a control (CTRL) subject. Panels A and C show data obtained at the end of the 14-day infusion (d14), while panels B and D show results obtained ≥ 1 month afterward (d45). Bars show means ± SEM. Dots show values in individual mice. Detailed statistical results are reported in Tables A8-A9.

## Slide 8
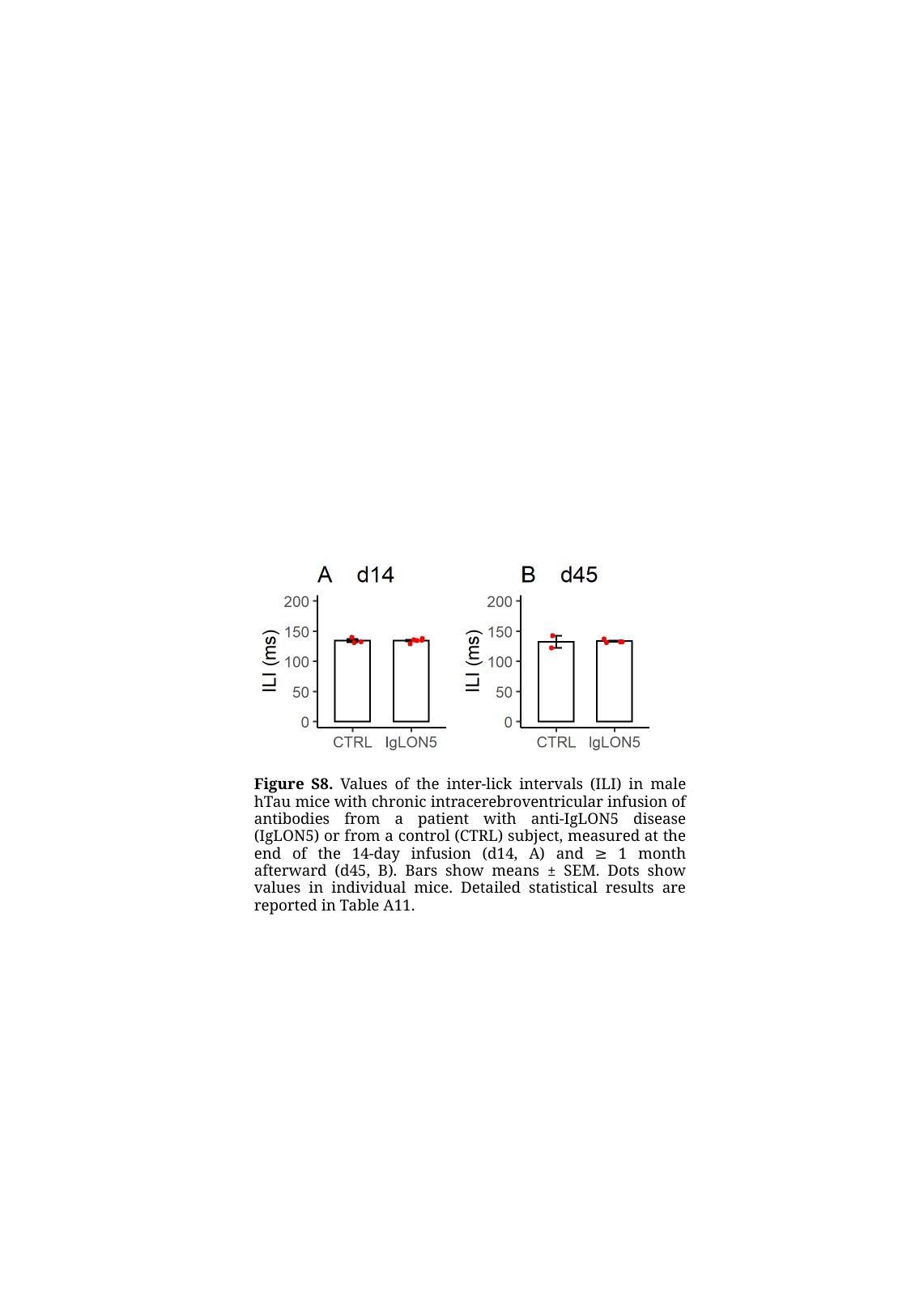

Figure S8. Values of the inter-lick intervals (ILI) in male hTau mice with chronic intracerebroventricular infusion of antibodies from a patient with anti-IgLON5 disease (IgLON5) or from a control (CTRL) subject, measured at the end of the 14-day infusion (d14, A) and ≥ 1 month afterward (d45, B). Bars show means ± SEM. Dots show values in individual mice. Detailed statistical results are reported in Table A11.
